# Massive scar contractures in unique presentation: Case report

**DOI:** 10.1016/j.ijscr.2023.108960

**Published:** 2023-10-13

**Authors:** Mohammed Abdulrazzak, Ghina Shehnah, Anwar Mohamad, Sounar Shehada, Aladdin Etr

**Affiliations:** a Faculty of Medicine, University of Aleppo, Aleppo, Syria; b CME Office, Faculty of Medicine, University of Aleppo, Aleppo, Syria; c Department of Plastic Surgery, Faculty of Medicine, Aleppo University, Aleppo, Syria

**Keywords:** Burn injuries, Contractures scar, Reconstruction, Case report, Syria

## Abstract

**Introduction:**

Scar contractures are a common complication of burn injuries, especially in the head and neck region. This paper presents a case of a middle-aged female who suffered severe scar contracture after a burn injury during the war in Syria.

**Presentation of case:**

A 33-year-old woman with a severe neck scar contracture resulting from a neglected burn injury presented to a plastic surgery department. The contractures extended to the chin, mandible, chest, and upper limbs. The patient underwent contracture release and reconstruction surgery, which involved the removal of the platysma and the placement of split-thickness skin grafts. The patient was discharged after one month of hospitalization. However, burn injuries require immediate and deliberate treatment, which may include reconstructive surgery.

**Discussion:**

Despite various efforts have been made to prevent the development of contractures, the contraction ratio of burn scars is still a badly controlled process, and reconstructive surgery is often indicated. There are many options to achieving the surgery, which vary in complexity. However, there is no preferable strategy and each option has advantages and disadvantages**.**

**Conclusion:**

Reconstructive is complete and technically demanded surgery, which needs special centers and professionals, this leads to poor results, especially in development countries like Syria.

## Highlights

• Scar contractures are a common complication for burns
• There is no preferable strategy for directing treatment.
• A 33-year-old woman admitted to plastic surgery department, with a sever neck scar contractures cover the neck and extended widely to the chin, mandible, chest and laterally to the upper limbs.

## 1 Introduction

Every year, burns cause over 7.1 million injuries, result in the loss of almost 18 million disability adjusted life years (DALYs), and contribute to more than 250,000 deaths worldwide. The low- and middle-income countries are particularly affected, especially in regions such as the Eastern Mediterranean, South East Asia, and Africa. Moreover, the majority of the burden of burn injury, more than 90 %, is experienced by low- and middle-income countries. Among these countries, the Eastern Mediterranean Region, the South East Asian Region, and the African Region bear the greatest burden, with the African Region alone accounting for nearly two-thirds of the total burden [1].

Scar contractures are a common complication that occurs after burns, when the burn scar thickens and tightens. It could limit joint movement, which will negatively impact day-to-day activities [2,3]. Unfortunately, the head and neck remain the most common in burn injuries that can lead to severe cervical contractures. Although every effort has been made to prevent the development of contractures, it remains poorly controlled and ends with surgery [4].

The treatment of a severe contraction is always difficult for the reconstructing surgeon. There is no preferable strategy for directing treatment, although a variety of surgical techniques have been described. In the event of cervical scarring, we must find an acceptable balance taking into consideration the functional and aesthetic aspects [5,6].

This paper shows a case of a middle-aged female, with severe neck scar contracture resulting from neglected, and careless burn injury in a unique presentation during the war in Syria, which underwent successful reconstructive surgery as per the SCARE 2020 Guidelines [7].

## 2 Presentation of case

A 33-year-old woman admitted to the plastic surgery department, with a severe neck scar contracture developed slowly over a year. 13 months earlier, she was exposed to a burn injury that affected most of her body while she was cooking, that treated conservatively and she later developed scar contractures. The patient was unable to access proper medical care in her area due to the war conditions, which led to this presentation.

**Fig. 1:**
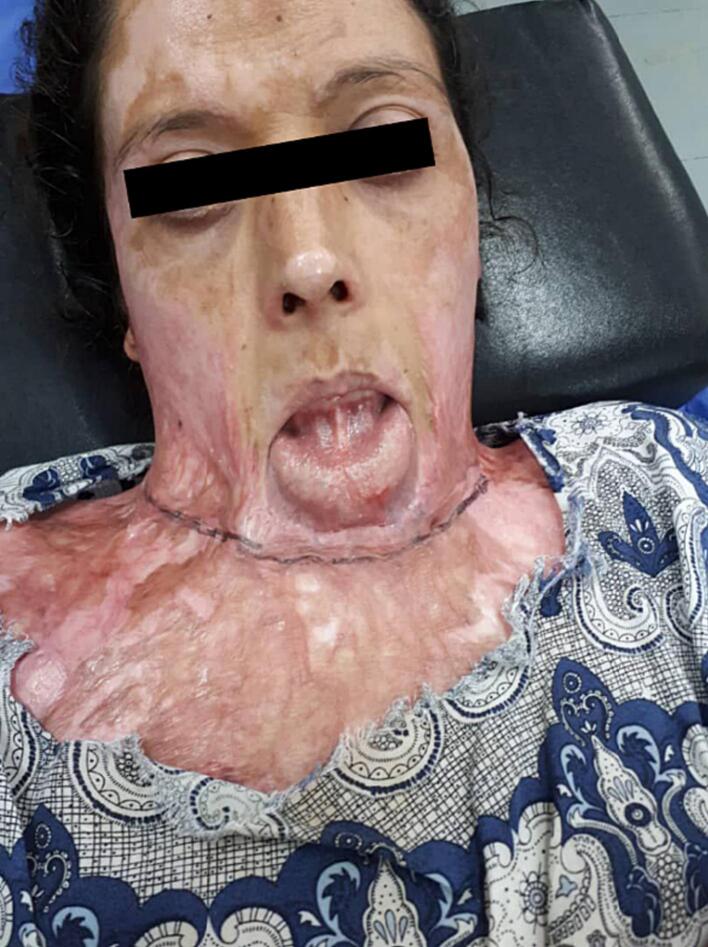
case presented; The contractures cover the neck, and extended widely to the chin, mandible, chest and laterally to the upper limbs.

The contractures cover the neck and extend widely to the chin, mandible, chest and laterally to the upper limbs. Although the burn covered a large area [Fig. 1], the damage did not exceed the superficial layer and did not affect deeper structures such as the trachea, nerves, and thyroid gland. The basic investigations show that the patient had anemia, she was continuously drooling and could not eat because of the shrinkage. There were no previous chronic diseases, no history of drug allergy, and no surgical history.

Laboratory tests including Complete Blood Count (CBC) show that the red blood cells (RBC) and hemoglobin (hg) were below the normal range. However, other blood tests, including Prothrombin time (PT), International Normalized Ratio (INR), Glucose test; Urea and Liver Function Tests (LFT) were within the normal limits.

**Fig. 2:**
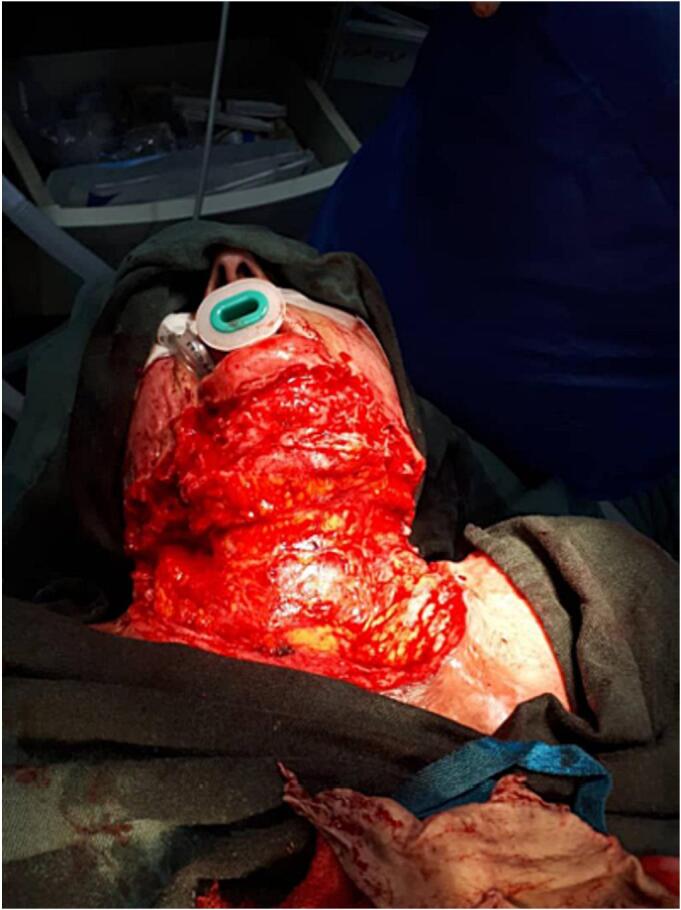
Hyperextension position, and platysma was carefully excised.

In light of our case, the patient underwent contracture release and reconstruction by a specialized team. Before the surgery, the excision site was precisely located and the graft was designed. During the surgery, the patient was placed on the operation table with a neck hyperextension position. Initially, the surgeon made a transverse incision on the scar along the width of the shrunken neck (due to difficulty in intubation). After that, the patient was intubated as shown in Fig. 2. The split-thickness skin grafts were taken from the skin of both thighs. The patient was kept in the hyperextension position, subsequently platysma was carefully excised due to the presence of large vessels, then the Split-thickness skin grafts were placed and fixed [Fig. 2].

**Fig. 3:**
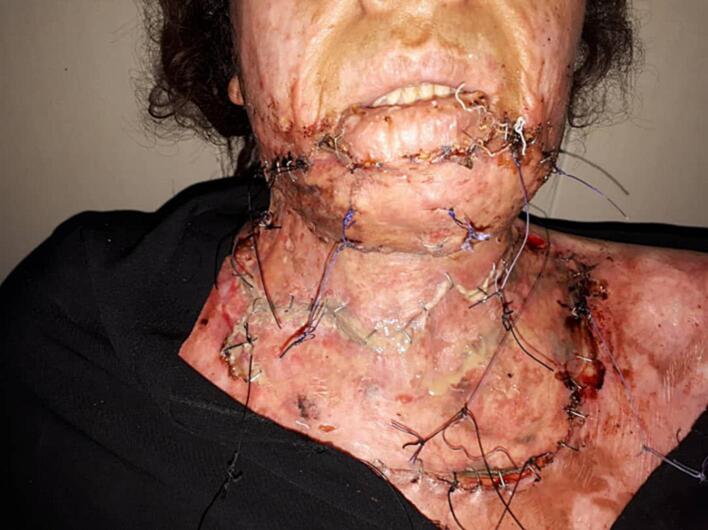
The affected area was bandaged and Fucidin ointment was applied on.

**Fig. 4:**
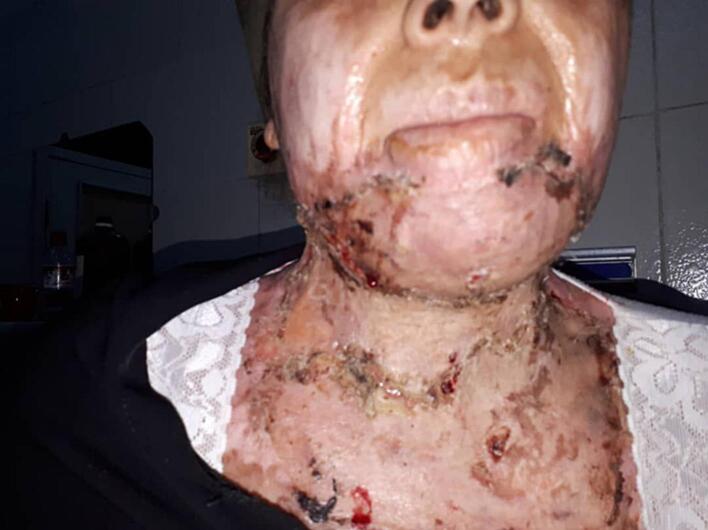
The first bandage was removed after 6 days.

**Fig. 5:**
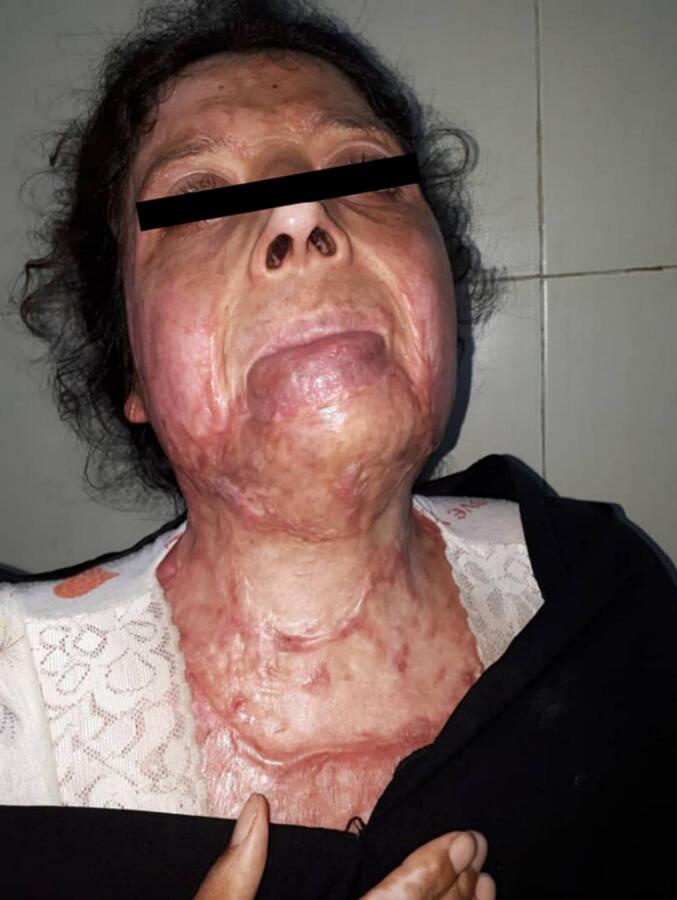
After cervical physiotherapy and follow up.

The Fucidin ointment was applied to the surgical site before the bandage implementation [Fig. 3]. The first bandage was removed after 6 days, after that a supportive cervical collar was put on [Fig. 4]. After a month, the patient was given cervical physiotherapy to improve the neck movement [Fig. 5].

The patient stayed for a month in the hospital and was discharged when her general condition became good. A follow-up of the case was not possible, as the patient did not return for routine follow-ups, and we could not contact her.

## 3 Discussion

The face and neck regions are the most common to be affected in severe burn injuries. The neck especially is an area with multidirectional activity, and post-burn scar contractures tend to form there easily. Furthermore, aesthetics aspects are particularly important in these areas [8]. Despite various efforts have been made to prevent the contractures development, the contraction ratio of burn scars is still a badly controlled process, and reconstructive surgery is often indicated. This implies that current methods or treatments for preventing or managing contractures in burn scars are not entirely effective, leading to the need for surgical intervention. However, most of the burden of born injuries is borne by middle- and low-income countries [1].

Several anatomical factors contribute to neck contractures, including the thin and smooth skin, the platysma muscle, cutaneous muscles, and flexion of the vertebral column, which is more pronounced in younger patients [9]. The severity of the scar formation is directly linked to the depth of the burn, the duration of healing, and re-epithelialization [2].

In the reported case, the patient suffered from a burn by the flame that was inadequately managed, leading to developing flexion contractures that restricted the range of joint movement and physical function beside the aesthetic deficits [10]. Ahuja and Bhattacharya in their systematic review highlight females aged 16–35 as being the most receptive to burn trauma as an outcome of their use of loose clothes, and their roles in cooking for the family, and they also emphasize flaming as a common cause of injuries [11].

In recent years, the field of burn reconstruction has been subject to ongoing debates and controversies. One of the key controversies revolves around the optimal timing of surgical intervention for neck contractures in burn patients. Some argue that early intervention is essential to prevent further scar contracture and functional limitations, while others advocate for a delayed approach to allow for tissue maturation and better surgical outcomes. This controversy highlights the need for further research and consensus among experts in the field [5].

Moreover, treating burn scar contractures poses a significant challenge for reconstructive surgeons. The primary goal of surgery is to release contractures and improve joint function by strategically incising the scar to maximize mobility. Prakash and Mullick highlight the difficulties of intubating patients with burn contractures, such as fixed flexion or changes in tracheal position. Therefore, in cases necessitating multiple surgical procedures, contracture release is typically performed first to ensure optimal airway control during subsequent surgeries. In the presented case, the surgeon made a transverse incision along the width of the neck scar to release the contractures and facilitate intubation [12].

There are many options to achieve wound healing that vary in complexity, such as full thickness grafts (FTG), split thickness skin grafts (STSG), V—Y pasties, V-M pasties, and free flaps. However, there is no preferable strategy and each option has advantages and disadvantages. Recent studies have focused on optimizing scar management strategies in burn reconstruction. These studies have explored the use of laser therapy, silicone gel sheets, and pressure garments to minimize scar formation and improve aesthetic outcomes. Additionally, advancements in wound healing research have led to the development of novel therapies, including growth factors and stem cell-based treatments, which hold promise for enhancing tissue regeneration and reducing scar contracture.

Skin grafts are the most common technique, because the issue in most patients resulted from skin loss. It is classified as full thickness or split thickness according to the amount of dermis in the graft [9]. full-thickness grafts provide superior color match and texture, better long-term durability, and are suitable for areas with limited mobility. However, they require a donor site and are more technically demanding. Split-thickness grafts have larger donor availability, quicker healing time, and flexibility in graft thickness, but they are less durable, have inferior color match and texture, and carry a risk of graft failure or necrosis. V—Y flaps offer larger tissue coverage, preserve blood supply, and allow for tension-free closure, but they require suitable adjacent tissue and have a more complex surgical technique. V-M flaps have similar advantages to V—Y flaps but can be used in different anatomical regions. Free flaps provide extensive tissue coverage and allow for the transfer of composite tissue, but they require specialized expertise, longer operative time, and have a higher risk of complications [13].

In the presented case, STSG harvested from the thighs was utilized to close the wound area. STSGs are commonly chosen when there is a need for rapid wound coverage and healing, especially in cases with limited donor availability or when the defect is relatively small. Additionally, STSGs are often preferred for areas with good vascularity, such as the limbs, as they have a higher chance of successful graft take. Potential complications of these approaches include hematoma, infection, bleeding, and necrosis. However, full-thickness grafts usually lead to secondary contractures, and their results are poor. One possible reason for the increased risk of contracture formation with full-thickness grafts is the difference in skin thickness and elasticity, which may contribute to a higher risk of contracture formation after full-thickness grafts [14].

Moreover, cognitive disorders are a significant issue in such cases and play an essential role in treatment of burn patients. The causes may include inflammation, damage to the blood-brain barrier, hormonal imbalances, and changes in nerve cells. To effectively reduce cognitive disorders after burns, it is important to focus on prevention and treatment strategies that target the specific mechanisms involved [15].

Following a successful surgery, the patient was closely monitored for a month and subsequently discharged when her overall condition improved. Unfortunately, she did not attend any routine follow-up appointments, and therefore, there are no further updates on her case.

## 4 Conclusion

The use of split-thickness skin grafts (STSGs) harvested from the thighs was chosen as a suitable approach for closing the wound area in the presented case. STSGs are commonly preferred for their ability to provide rapid wound coverage and healing, especially in cases with limited donor availability or smaller defects. The success of STSGs is often higher in areas with good vascularity, such as the limbs. However, potential complications, including hematoma, infection, bleeding, and necrosis, should be considered. The field of burn reconstruction is continuously evolving, and new techniques and discoveries are being made. However, during the literature search conducted for this discussion, no groundbreaking discoveries in burn reconstruction for neck contractures were found in recent years. It is always recommended to consult with a qualified plastic surgeon or burn specialist to obtain the most up-to-date information on advancements and techniques in this field.

## Ethical approval

Not applicable.

## Funding

There are no funding sources.

## CRediT authorship contribution contribution

The work's conception and design: all authors.

paper writing, and article revision: all authors.

Final revision and approval: all authors.

## Guarantor

Aladdin Etr.

## Registration of research studies

Research Registry

researchregistry9044

https://www.researchregistry.com/browse-the-registry#home/registrationdetails/6467e0e6e6cf62002a8f6af5/

## Declaration of competing interest

The authors declare that they have no competing interests.

## Data Availability

Not applicable.
